# Multiplex, Rapid, and Sensitive Isothermal Detection of Nucleic-Acid Sequence by Endonuclease Restriction-Mediated Real-Time Multiple Cross Displacement Amplification

**DOI:** 10.3389/fmicb.2016.00753

**Published:** 2016-05-18

**Authors:** Yi Wang, Yan Wang, Lu Zhang, Dongxin Liu, Lijuan Luo, Hua Li, Xiaolong Cao, Kai Liu, Jianguo Xu, Changyun Ye

**Affiliations:** ^1^State Key Laboratory of Infectious Disease Prevention and Control, National Institute for Communicable Disease Control and Prevention, Collaborative Innovation Center for Diagnosis and Treatment of Infectious Diseases, Chinese Center for Disease Control and PreventionBeijing, China; ^2^Pathogenic Biology Institute, University of South ChinaHengyang, China; ^3^Department of Microbiology, Guiyang Medical UniversityGuiyang, China

**Keywords:** ET-MCDA, MCDA, LoD, *Salmonella* spp., *Shigella* spp.

## Abstract

We have devised a novel isothermal amplification technology, termed endonuclease restriction-mediated real-time multiple cross displacement amplification (ET-MCDA), which facilitated multiplex, rapid, specific and sensitive detection of nucleic-acid sequences at a constant temperature. The ET-MCDA integrated multiple cross displacement amplification strategy, restriction endonuclease cleavage and real-time fluorescence detection technique. In the ET-MCDA system, the functional cross primer E-CP1 or E-CP2 was constructed by adding a short sequence at the 5′ end of CP1 or CP2, respectively, and the new E-CP1 or E-CP2 primer was labeled at the 5′ end with a fluorophore and in the middle with a dark quencher. The restriction endonuclease Nb.*BsrDI* specifically recognized the short sequence and digested the newly synthesized double-stranded terminal sequences (5′ end short sequences and their complementary sequences), which released the quenching, resulting on a gain of fluorescence signal. Thus, the ET-MCDA allowed real-time detection of single or multiple targets in only a single reaction, and the positive results were observed in as short as 12 min, detecting down to 3.125 fg of genomic DNA per tube. Moreover, the analytical specificity and the practical application of the ET-MCDA were also successfully evaluated in this study. Here, we provided the details on the novel ET-MCDA technique and expounded the basic ET-MCDA amplification mechanism.

## Introduction

Dramatic growth of available sequence information has made the specific detection of nucleic acids critical to the development of the modern biology sciences (Guo et al., 2009). Among the many technologies developed for the specific detection and analysis of nucleic acids, sequence-based amplification is one of the most important process and underpins the modern molecular biology (Li and Macdonald, 2015). The polymerase chain reaction (PCR) provides the most versatile technique to detect trace quantities of genes or nucleotide sequences present in sample materials (Letchumanan et al., 2014; Wang et al., 2015a). However, PCR and its derivatives (such as real-time PCR and nested PCR) could not get rid of the limitation of thermal cycling steps for successful amplification, and the resultant instrumental restraint has been hampering the uptake of PCR-based analysis in point-of-use, field settings and more (Craw and Balachandran, 2012). Hence, the establishment of alternative methodologies for the simple, rapid and specific analysis of nucleic acid is in continuous demand.

Accordingly, dozens of isothermal nucleic acid amplification technologies have been devised by various laboratories in the past 20 years, which eliminated the use of a costly specialized apparatus, and exhibited high amplification efficiency comparable to the that of PCR (Zhao et al., 2015). These mainly include nucleic acid sequence-based amplification (NASBA), self-sustained sequence replication reaction (3SR), strand displacement amplification (SDA), exponential amplification reaction (EXPAR), helicase-dependent amplification (HDA), recombinase polymerase amplification (RPA), single primer isothermal amplification (SPIA), rolling circle amplification (RCA), loop-mediated isothermal amplification (LAMP), and cross-priming amplification (CPA; Deng and Gao, 2015; Zhao et al., 2015). Within these isothermal amplification techniques, NASBA, 3SR, SDA, EXPAR, HDA, RPA, and SPIA are still relatively complex protocols requiring multiple enzymes (two or more), rigorous optimization and/or special reagents (Zhao et al., 2015). Only a few of these assays (e.g., RCA, LAMP, and CPA) can be efficiently carried out at a fixed temperature using one enzyme (Zhao et al., 2015). However, RCA was restricted to amplify the circular target DNA, limiting the usefulness of the technique (Ali et al., 2014). Although LAMP and CPA methodologies have been verified to be useful for basic, clinical and applied research, the trace amounts of target sequences were still difficultly to detect in various samples (Niemz et al., 2011; Wang et al., 2014, 2015c).

In a recent study, a novel isothermal nucleic acid amplification technique, named multiple cross displacement amplification (MCDA), was devised to overcome the technical difficulties posed by current isothermal nucleic acid amplification methodologies and offered advantages on simplicity, sensitivity, specificity, time consumption and easiness in operation (Wang et al., 2015d). In order to be more valuable and widely applied in modern medicine and biology, the ability to simultaneously analyze multiple different targets in a single sample will be extremely important. For achieving multiplex MCDA-based detection, we devised the novel multiplex MCDA method we called endonuclease restriction-mediated real-time multiple cross displacement amplification (ET-MCDA). The ET-MCDA assay combines endonuclease restriction and real-time fluorescence detection with MCDA approach, which can simultaneously detect multiple targets in a single reaction in approximately 12 min. Here, we expound the basic ET-MCDA principle and demonstrate an application of the novel methodology.

## Materials and methods

### Reagents

Nb.*BsrDI* was purchased from New England BioLabs (Beijing, China). The DNA Extraction Kits (QIAamp DNA Mini kits) were purchased from Qiagen (Beijing, China). The Loopamp™ Flourescent Detection Reagent (FD) and Loopamp Kits were purchased from Eiken Chemical Co. Ltd. (Beijing, China).

### ET-MCDA primers

**Figure 1:**
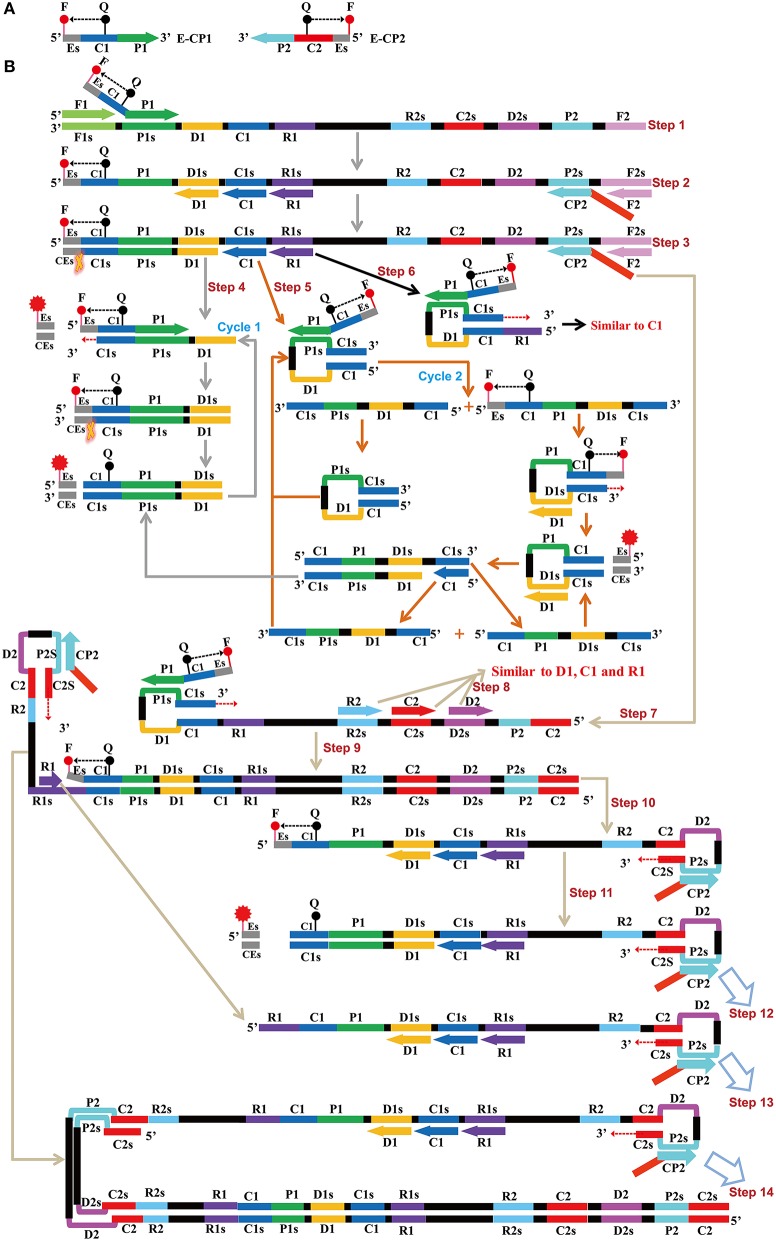
**Mechanistic description of the ET-MCDA amplification. (A)** Schematic depiction of a new cross primer (E-CP1 or E-CP2). E-CP1/E-CP2, which was an extension of the cross primer CP1 (C1+P1)/CP2 (C2+P2) with a restriction endonuclease recognition site (Es, its complementary sequence site termed CEs) at the 5′ end, was modified with a fluorophore (F) at the 5′ end and a quencher (Q) in the middle. **(B)** The outline of ET-MCDA amplification. Step 1: The E-CP1 initiates the ET-MCDA amplification at the P1s site of target sequence, and the newly synthesized strand will be displaced by the upstream synthesis from the F1 primer. Step 2: Three amplification primers (D1, C1, and R1), cross primer (CP2) and displacement primer (F2) anneal to the newly synthesized strand, and the *Bst* polymerase extends in tandem generating four products. Step 3: The new double-stranded terminal sequence (Es and the complementary CEs sequences) was digested by restriction endonuclease (Nb.*BsrDI*), resulting a gain of fluorescence signal. Step 4: The D1 product is displaced by the synthesis from amplification primer C1, and the resulting structure undergoes the cycling amplification step (Cycle 1). Thus, this process releases the quenching, resulting the additional again of signal. Step 5, 6: Similar to D1 product, the C1 and R1 products initiate additional two cycles, and the more fluorescence signals are obtained in these processes. Step 7, 9, 10, 11, 12, 13, 14: The products from steps 7 to 14 serve as the templates for subsequent elongation and cycling amplification steps, which give rise to additional release of quenching, resulting in exponential signal detection. Moreover, the D2, C2, and R2 products (Step 8) will undergo three cycling amplification process, which are similar to D1, C1, and R1 products.

**Figure 2:**
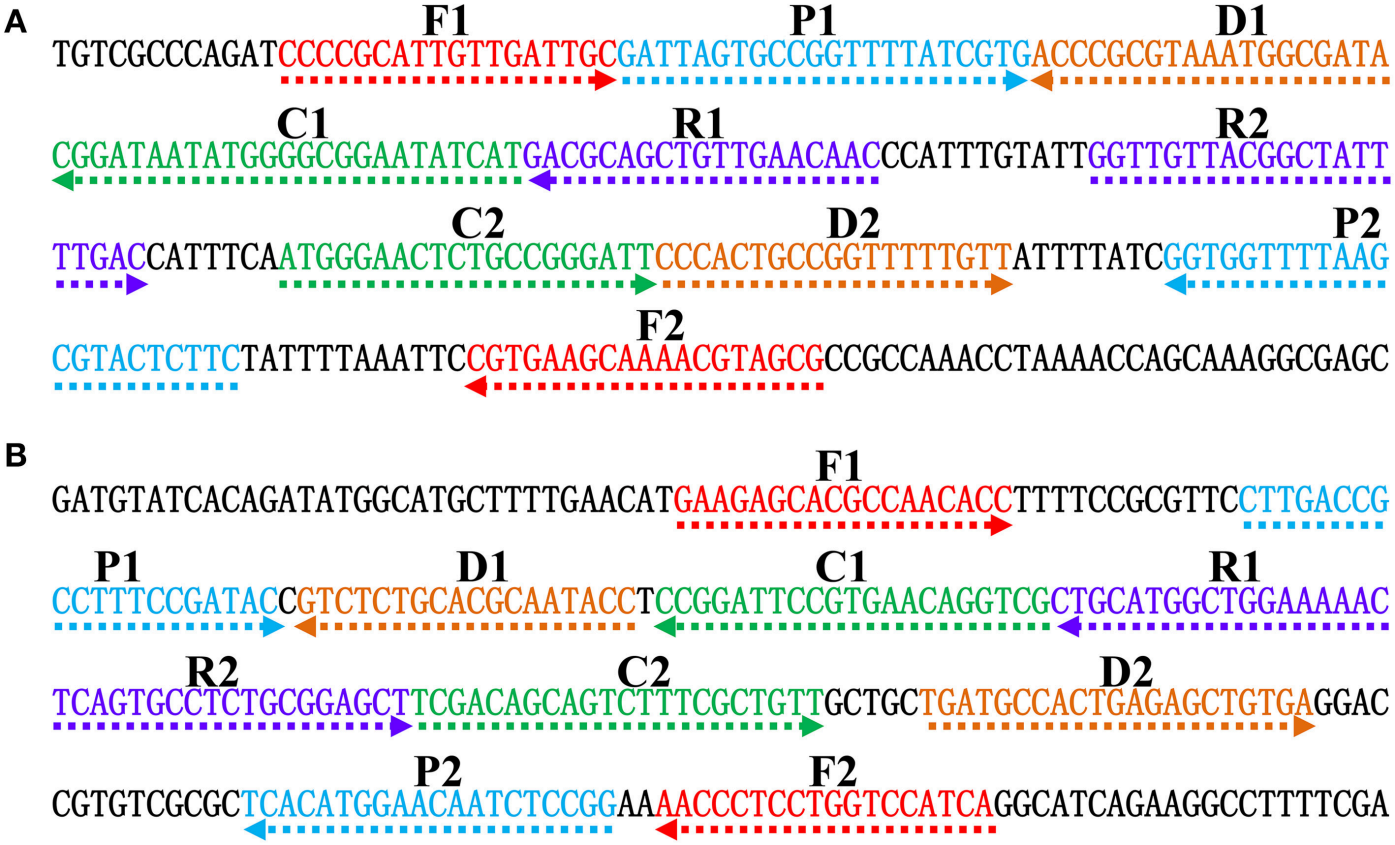
**Nucleotide sequence and location of *Salmonella* (*invA*) and *Shigella* (*ipaH*) genes used to design ET-MCDA primers**. The nucleotide sequences of the sense strands of *invA* **(A)** and *ipaH* **(B)** are listed. The sites of primer sequences were underlined. Left arrows and right arrows showed complementary and sense sequences that are used.

The *Salmonella* and *Shigella* were selected as model target microorganisms for validating the usability of ET-MCDA technology. Two specific genes (*invA, Salmonella* spp.-specific gene; *ipaH, Shigella* spp.-specific gene) were chosen as the target sequences for designing ET-MCDA primers (Galan et al., 1992; Gaudio et al., 1997). Based on the *invA* gene of *Salmonella* spp. and *ipaH* gene of *Shigella* spp., two sets of ET-MCDA primers were designed by primer software PRIMER PREMIER 5.0 and PrimerExplorer V4 (Eiken Chemical, Japan) according to the mechanism of ET-MCDA (Figure 1). Blast analysis ascertained that two sets of ET-MCDA primers were specific for *Salmonella* spp. strains and *Shigella* spp. strains. The details of primer design, primers sequences and locations of ET-MCDA primers were listed in Figure 2 and Table 1. The dark quencher used was Black Hole Quencher-1 and Black Hole Quencher-2 (Biosearchtech, Shanghai, China), and the fluorophores used were Cy3 and HEX, which can be detected in a real-time system that was used for conducting the ET-MCDA reactions. All of the oligomers were synthesized and purified by Tianyi-Biotech (Beijing, China).

**Table 1:** **The primers used in the study**.

**Primers name^a^**	**Sequences and modifications**	**Length**	**Genes^b^**
Sal-F1	5′-CCCCGCATTGTTGATTGC-3′	18 nt	
Sal-F2	5′-CGCTACGTTTTGCTTCACG-3′	19 nt	
Sal-CP1	5′-ATGATATTCCGCCCCATATTATCCG-GATTAGTGCCGGTTTTATCGTG-3′	47 mer	
Sal-E-CP1	5′-Cy5-TGCAATG-ATGATAT(BHQ2)TCCGCCCCATATTATCCGGATTAGTGCCGGTTTTATCGTG-3′	54 mer	
Sal-CP2	5′-ATGGGAACTCTGCCGGGATT-GAAGAGTACGCTTAAAACCACC-3′	42 mer	
Sal-C1	5′-ATGATATTCCGCCCCATATTATCCG-3′	25 nt	*invA*
Sal-C2	5′-ATGGGAACTCTGCCGGGATT-3′	20 nt	
Sal-D1	5′-TATCGCCATTTACGCGGGT-3′	19 nt	
Sal-D2	5′-GTTGTTCAACAGCTGCGTC-3′	19 nt	
Sal-R1	5′-CCCACTGCCGGTTTTTGTT-3′	19 nt	
Sal-R2	5′-GGTTGTTACGGCTATTTTGAC-3′	21 nt	
Shi-F1	5′-GAAGAGCACGCCAACACC-3′	18 nt	
Shi-F2	5′-TGATGGACCAGGAGGGTT-3′	18 nt	
Shi-CP1	5′-CGACCTGTTCACGGAATCCGG-CTTGACCGCCTTTCCGATAC-3′	41 mer	
Shi-E-CP1	5′-Hex-TGCAATG-CGACCT(BHQ1)GTTCACGGAATCCGGCTTGACCGCCTTTCCGATAC-3′	48 mer	
Shi-CP2	5′-TCGACAGCAGTCTTTCGCTGTT-CCGGAGATTGTTCCATGTGA-3′	42 mer	
Shi-C1	5′-CGACCTGTTCACGGAATCCGG-3′	21 nt	*ipaH*
Shi-C2	5′-TCGACAGCAGTCTTTCGCTGTT-3′	22 nt	
Shi-D1	5′-GGTATTGCGTGCAGAGAC-3′	18 nt	
Shi-D2	5′-GTTTTTCCAGCCATGCAG-3′	18 nt	
Shi-R1	5′-TGATGCCACTGAGAGCTGTGA-3′	21 nt	
Shi-R2	5′-TCAGTGCCTCTGCGGAGCT-3′	19 nt	

a *Sal, salmonella; Shi, shigella*.

b *mer, monomeric unit; nt, nucleotide*.

### Bacterial strains

A total of 55 strains used in this study were listed in Table 2. These strains were stored in 10% (w/v) glycerol BHI broth at −70°C. All strains were refreshed three times on nutrient agar plate at 37°C, and then were applied to enrich and extract genomic DNA templates. Moreover, the *Salmonella enterica* serovar *Enteritidis* (ICDC-NPSa003) and *Shigella flexneri* serotype 4av (ICDC-NPS007) strains were selected for the rest of confirmation performance, sensitivity analysis, optimal temperature and practical application conducted in this study.

**Table 2:** **Bacterial strains used in this study**.

**Bacteria**	**Serovar/species**	**Strain no. (source of strains)^a^**	**No. of strains**
*Salmonella enterica*	Choleraesuis	ICDC-NPSa001	1
	Dublin	ICDC-NPSa002	1
	Enteritidis	ICDC-NPSa003	4
	Typhimurium	ICDC-NPSa004	2
	Weltevreden	ICDC-NPSa005	1
	U	Isolated strains (ICDC)	6
*Shigella flexneri*	1d	ICDC-NPS001	1
	4a	ICDC-NPS002	1
	5a	ICDC-NPS003	1
	2b	ICDC-NPS004	1
	1b	ICDC-NPS005	1
	3a	ICDC-NPS006	1
	4av	ICDC-NPS007	1
	3b	ICDC-NPS008	1
	5b	ICDC-NPS009	1
	Y	ICDC-NPS0010	1
	Yv	ICDC-NPS0011	1
	Y	ICDC-NPS0012	1
	X	ICDC-NPS0013	1
	Xv	ICDC-NPS0014	1
*Shigella sonneri*	U	Isolated strains (ICDC)	2
*Shigella dysenteriae*	U	Isolated strains (ICDC)	2
*Shigella boydii*	U	Isolated strains (ICDC)	1
*Shigella flexneri*	1d	ICDC-NPS001	1
*Listeria monocytogenes*	1/2a	EGD-e	1
	4c	ATCC19117	1
*Enterohemorrhagic E. coli*	U	EDL933	1
	U	Isolated strains (ICDC)	1
*Enteroaggregative E. coli*	U	Isolated strains (ICDC)	1
*Enterotoxigenic E. coli*	U	Isolated strains (ICDC)	1
*Enteroinvasive E. coli*	U	Isolated strains (ICDC)	1
*Enteropathogenic E. coli*	U	Isolated strains (ICDC)	1
*Plesiomonas shigelloides*	U	ATCC51903	1
*Enterobacter cloacae*	U	Isolated strains (ICDC)	1
*Enterococcus faecalis*	U	ATCC35667	1
*Yersinia enterocolitica*	U	ATCC23715	1
*Bntorobater sakazakii*	U	Isolated strains (ICDC)	1
*Vibrio cholerae*	U	Isolated strains (ICDC)	1
*Vibrio parahaemolyticus*	U	Isolated strains (ICDC)	1
*Vibrio vulnificus*	U	Isolated strains (ICDC)	1
*Staphylococcus aureus*	U	Isolated strains (ICDC)	1
*Campylobacter jejuni*	U	ATCC33291	1
*Bacillus cereus*	U	Isolated strains (ICDC)	1
*Pseudomonas aeruginosa*	U	Isolated strains (ICDC)	1

a *U, unidentified serotype; ATCC, American Type Culture Collection; ICDC, National Institute for Communicable Disease Control Disease Control and Prevention, Chinese Center for Disease Control and Prevention*.

### Genomic DNA extraction

According to the manufacturer's instructions, bacterial genomic templates were extracted from all culture strains using DNA kits (QIAamp DNA Mini Kits; Qiagen, Hilden, Germany). The concentrations of extracted DNA templates were examined with ultraviolet spectrophotometer at A260/280 (Nano drop ND-1000, Calibre, Beijing, China). The DNA samples were stored at −20°C before they were used.

### The MCDA reaction

In order to evaluate the utility of two sets of MCDA primers, the MCDA assay either for *Salmonella* spp. strains or *Shigella* spp. strains was carried out as the following description. Briefly, The MCDA reaction was conducted with the Loopamp DNA amplification Kit in a final volume of 25 μl conitaining 0.4 μM each of displacement primers F1 and F2, 0.8 μM each of amplification primers C1 and C2, 1.2 μM each of amplification primers R1, R2, D1, and D2, 2.4 μM each of cross primers CP1 and CP2, 12.5 μl 2 × reaction mix, 1 μl FD, 1.25 μl of *Bst* DNA polymerase (10 U) and 1 μl DNA template (250 pg/μl).

The reaction mixtures of normal MCDA were incubated at 63°C for 1 h and then heated at 85°C for 5 min to stop the reaction. A total of three methods were used for analyzing the normal MCDA amplification. The color change of positive reactions in MCDA tubes from light gray to green could be directly observed by FD reagent, and the MCDA products were also monitored by electrophoresis on 2% agarose gels with ethidium bromide staining. Furthermore, real-time monitoring of normal MCDA amplifications was carried out by recording the optical density (OD) at 650 nm every 6 s using the Loopamp Real-time Turbidimeter LA-320C (Eiken Chemical Co., Ltd, Japan). A positive reaction was density as a threshold value of > 0.1 within 60 min and analysis of each dilution (sample) was examined at least two times. The reaction mixtures without templates were selected as a negative control.

### The standard ET-MCDA reaction

To further evaluate the availability of two sets of ET-MCDA primers, the ET-MCDA approach either for *Salmonella* spp. strains or *Shigella* spp. strains was conducted as the following description. The reaction mixtures of standard ET-MCDA were carried out with the Loopamp DNA amplification Kit in a final volume of 25 μl containing 0.4 μM each of displacement primers F1 and F2, 0.8 μM each of amplification primers C1 and C2, 1.2 μM each of amplification primers R1, R2, D1, and D2, 1.2 μM E-CP1 and CP1 primers, 2.4 μM CP2 primers, 12.5 μl 2 × reaction mix, 1.25 μl of *Bst* DNA polymerase (10 U), 1.5 μl (15 U) of Nb.*BsrDI* endonuclease and 1 μl DNA template.

The amplification mixtures were performed at 63°C for 1 h and then incubated at 85°C for 5 min to stop the reaction. Mixtures without DNA template were chosen as a negative control. After amplification, the ET-MCDA products were analyzed by electrophoresis on 2% agarose gels with ethidium bromide staining or directly observed the color change by FD reagent. Furthermore, the ET-MCDA amplifications were monitored by real-time detection.

### The optimal reaction temperature of ET-MCDA method

In order to assess the optimal amplification temperature, the ET-MCDA reaction mixtures were conducted at a constant temperature ranging from 60 to 65°C for 1 h and then incubated at 85°C for 5 min to stop the reaction. Mixtures without the template were selected as a negative control.

### The multiplex ET-MCDA method

For multiplex detections, the amplification mixtures of multiplex ET-MCDA conducted with the Loopamp DNA amplification Kit in a final volume of 25 μl containing 0.4 μM each of displacement primers *Sal*-F1 and *Sal*-F2, 0.2 μM each of amplification primers *Sal*-C1 and *Sal*-C2, 1.2 μM each of amplification primers *Sal*-R1, *Sal*-R2, *Sal*-D1, and *Sal*-D2, 1.2 μM *Sal*-E-CP1 and *Sal*-CP1 primers, 2.4 μM *Sal*-CP2 primers, 0.4 μM each of displacement primers *Shi*-F1 and *Shi*-F2, 0.2 μM each of amplification primers *Shi*-C1 and *Shi*-C2, 0.32 μM each of amplification primers *Shi*-R1, *Shi*-R2, *Shi*-D1, and *Shi*-D2, 0.32 μM *Shi*-E-CP1 and *Shi*-CP1 primers, 0.64 μM *Shi*-CP2 primers, 12.5μl 2 × reaction mix, 1.25 μl of *Bst* DNA polymerase (10 U), 1.5 μl (15 U) of Nb.*BsrDI* endonuclease and 1 μl each DNA template of *Salmonella* spp. strains and *Shigella* spp. strains. The ET-MCDA mixtures were performed at 63°C for 60 min in a Rotor-Gene Q Real Time System (Qiagen) and mixtures without genomic DNA template was used as a negative control. Three replicates of the lowest detectable template amount were tested.

### Evaluation of sensitivity of the ET-MCDA method

In order to make a comparative analysis of the ET-MCDA, MCDA, quantitative PCR (qPCR) and PCR approaches, the genomic DNA templates of strains ICDC-NPSa003 (*Salmonella Enteritidis*) and ICDC-NPS007 (*Shigella flexneri*) were serially diluted to determine the limit of detection (LoD). The LoD of ET-MCDA, MCDA, qPCR, and PCR assays was defined by genomic DNA amount of the template. The lowest detectable template amount was tested in triplicate.

### Evaluation of specificity of the multiplex ET-MCDA method

In order to evaluate the analytical specificity of ET-MCDA technology, the multiplex ET-LAMP reactions were carried out under the conditions described above with purely genomic templates from 15 *Salmonella* strains, 20 *Shigella* strains, 20 non- *Salmonella*, and non-*Shigella* strains (Table 2). Analysis of each sample was performed in at least two independent experiments.

### Practical application of ET-MCDA to *Salmonella* and *Shigella* detection in human blood samples

The human blood samples were acquired from a healthy donor with the written informed consent. Our study was reviewed and approved by the ethics committee of the National Institute for Communicable Disease Control and Prevention, China CDC, according to the medical research regulations of the Ministry of Health China (Approval No. ICDC2014003).

In order to assess the practicability of ET-MCDA technology, the novel ET-MCDA technique was applied to identify and differentiate the target pathogens in human blood samples. The human blood samples were confirmed as being *Salmonella*- and *Shigella*-negative by conventional culture techniques and PCR assays. *Salmonella enterica* serovar *Enteritidis* (ICDC-NPSa003) and *Shigella flexneri* serotype 4av (ICDC-NPS007) were simultaneously placed into the blood samples.

The artificially contaminated blood samples were performed as previous study (Wang et al., 2015b). Firstly, to test the minimal detectable colony forming units (CFUs), the cultures with *Salmonella Enteritidis* or *Shigella flexneri* strains were serially diluted (10^−1^–10^−9^), and the aliquots of 100 μl appropriate dilution (10^−6^) were spread onto brain heart infusion (BHI) agar in triplicate. Then, the numbers of the inocula were counted after incubation at 37°C for 24 h. The following steps, the aliquots of 100 μl appropriate dilutions with *Salmonella Enteritidis* and *Shigella flexneri* were simultaneously added to the blood samples, and the numbers of *Salmonella* were adjusted to approximate 3.9 × 10^6^, 3.9 × 10^5^, 3.9 × 10^4^, 3.9 × 10^3^, 3.9 × 10^2^, 3.9 × 10^1^, and 3.9 × 10^0^ CFU/ml, *Shigella* for 3.4 × 1 0^6^, 3.4 × 10^5^, 3.4 × 10^4^, 3.4 × 10^3^, 3.4 × 10^2^, 3.4 × 10^1^, and 3.4 × 10^0^ CFU/ml. Simultaneously, aliquots (100 μl) of the artificially contaminated blood were applied to extract genomic DNA templates, and the supernatants (1 μl) were used for multiplex ET-MCDA, MCDA, qPCR and conventional PCR test. Non-contaminated blood samples were chosen as negative control and performances were conducted in triplicate independently.

## Results

### The ET-MCDA design

The ET-MCDA design and amplification scheme is depicted in Figure 1. The novel ET-MCDA technique integrates isothermal amplification method, real-time and fluorescence detection technology. In the ET-MCDA system, the cross primer CP1 or CP2 contains a 5′ end short sequence (Es) that can be recognized by restriction endonuclease Nb.*BsrDI*, and the novel cross primers are termed E-CP1 or E-CP2. The novel E-CP1 or E-CP2 is modified at 5′ end with a fluorophore and in the middle with a corresponding dark quencher (Figure 1A). One fluorophore is assigned to one target primer set, thus the ET-MCDA assay allows for real-time detection of multiple, distinct targets in a constant reaction using the novel E-CP1or E-CP2 primer.

The restriction endonuclease Nb.*BsrDI*, which can recognize the sequence 5′-GCAATGNN-3′ (N = G, C, A, and T) and digests target sequence 5′-GCAATG-3′ at a single temperature from 60 to 65°C, is selected for the ET-MCDA reaction. In order to construct E-CP1 and E-CP2 primers, the nucleotide sequence 5′-GCAATG-3′ can be added to the 5′ end of any CP1 and CP2 primers, and the novel E-CP1 or E-CP2 maintains its function as a MCDA primer with the added advantage of simultaneous real-time determination of the ET-MCDA reaction by release of quenching (Figure 1B). Herein, the ET-MCDA amplification, cleaving and real-time detection can be simultaneously conducted at a single temperature.

### The primer design for the ET-MCDA technology

In order to demonstrate the mechanism of ET-MCDA assay, the *invA* gene (GenBank accession no. NC. 003197) and the *ipaH* gene (GenBank accession no. M32063), which were specific for *Salmonella* spp. and *Shigella* spp. strains, were chosen as the model targets. Two ET-MCDA primer sets, which recognized 10 regions more than 200 base pairs on *invA* gene and *ipaH* gene, were designed by using the PrimerExplorer V4 (Eiken Chemical) and primer software PRIMER PREMER 5.0 according to the principle of ET-MCDA technique. The specificity of the two ET-MCDA primer sets was examined by using the NCBI (Basic Local Alignment Search Tool), and no exact match against non-*Shigella* spp. and non-*Salmonella* spp. strains. The details of the target sequences, primer design, location and sequences were presented in Figure 2 and Table 1.

### Confirm and detection of *Salmonella*- and *Shigella*-MCDA products

**Figure 3:**
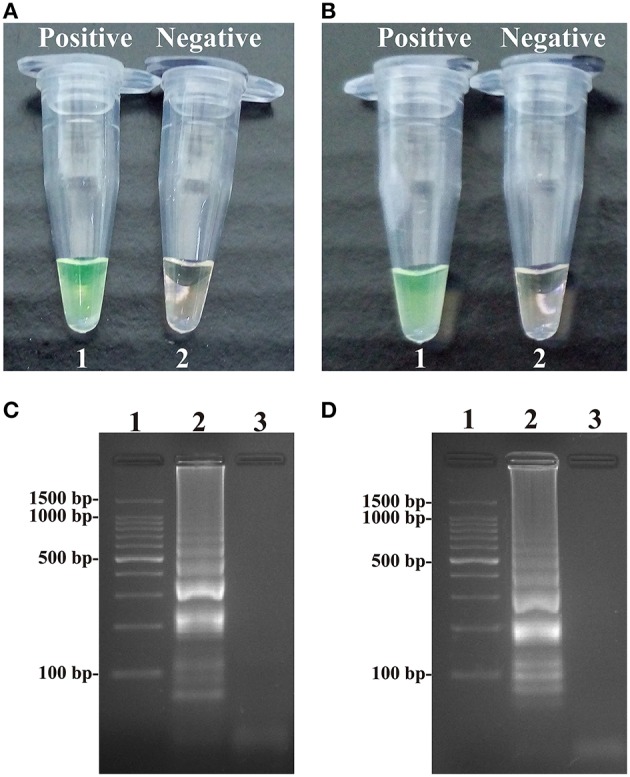
**Detection and confirmation of *Salmonella*- and *Shigella*-MCDA products. (A,B)** Color change of *Salmonella*- and *Shigella*-MCDA tubes; tube 1, positive amplification; tube 2, negative amplification. **(C,D)** 2% agarose gel electrophoresis applied to *Salmonella*- and *Shigella*-MCDA products; lane 1, DL 100-bp DNA marker; lane 2, positive MCDA reaction, lane 3, negative MCDA reaction.

A color shift of positive reactions in *Salmonella*- and *Shigella*-MCDA tubes from light gray to green was directly seen with naked eyes within 1 h incubation periods at 63°C (Figures 3A,B). The MCDA products were also analyzed by 2% agarose gel electrophoresis, and the typical ladder-like patterns were visible (Figures 3C,D). Thus, two MCDA primer sets for *Salmonella* and *Shigella* detection were good candidates for establishment of the ET-MCDA approaches.

### Confirm and detection of ET-MCDA products in nonreal-time format

**Figure 4:**
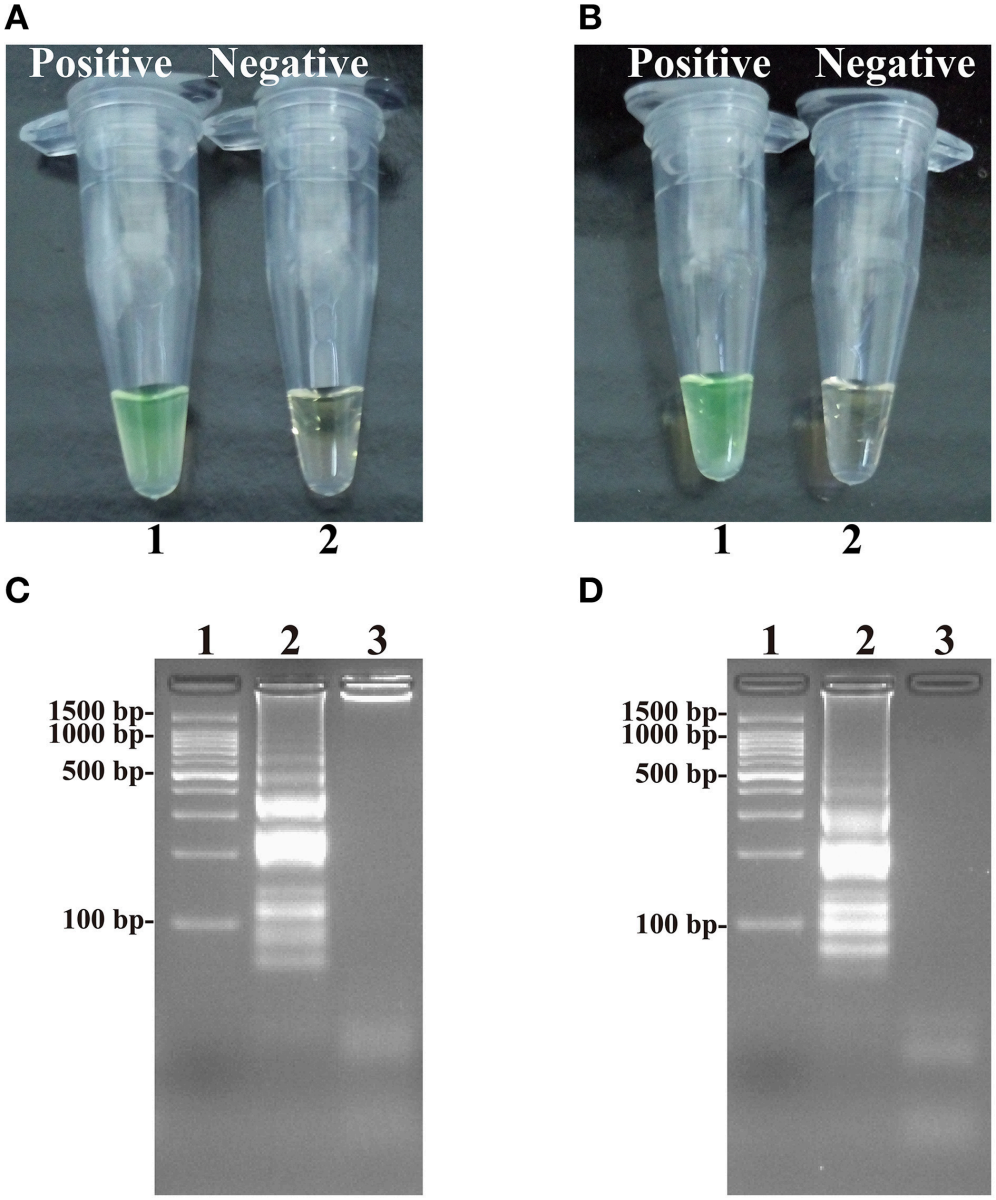
**Detection and confirmation of *Salmonella*- and *Shigella*-ET-MCDA products in Nonreal-time format. (A,B)** Color change of *Salmonella*- and *Shigella*-ET-MCDA tubes; tube 1, positive amplification; tube 2, negative amplification. **(C,D)** 2% agarose gel electrophoresis applied to *Salmonella*- and *Shigella*-ET-MCDA products; lane 1, DL 100-bp DNA marker; lane 2, positive ET-MCDA reaction, lane 3, negative ET-MCDA reaction.

At 63°C, the ET-MCDA amplifications were carried out in the presence or absence genomic DNA templates according to the standard ET-MCDA condition. The ET-MCDA amplifications were directly detected in-tube by color transition of FD reagent from light gray to green, which were visible to the naked eye (Figures 4A,B). Then, the ET-MCDA products were also analyzed by 2% agarose gel electrophoresis, and the typical ladder-like patterns were observed but not in negative control (Figures 4C,D). These results indicated that the novel ET-MCDA strategy could correctly amplification target sequences and the *invA*- and *ipaH*-ET-MCDA primers were suitable for *Salmonella* and *Shigella* detection.

### The optimal reaction temperature of the ET-MCDA assay

**Figure 5:**
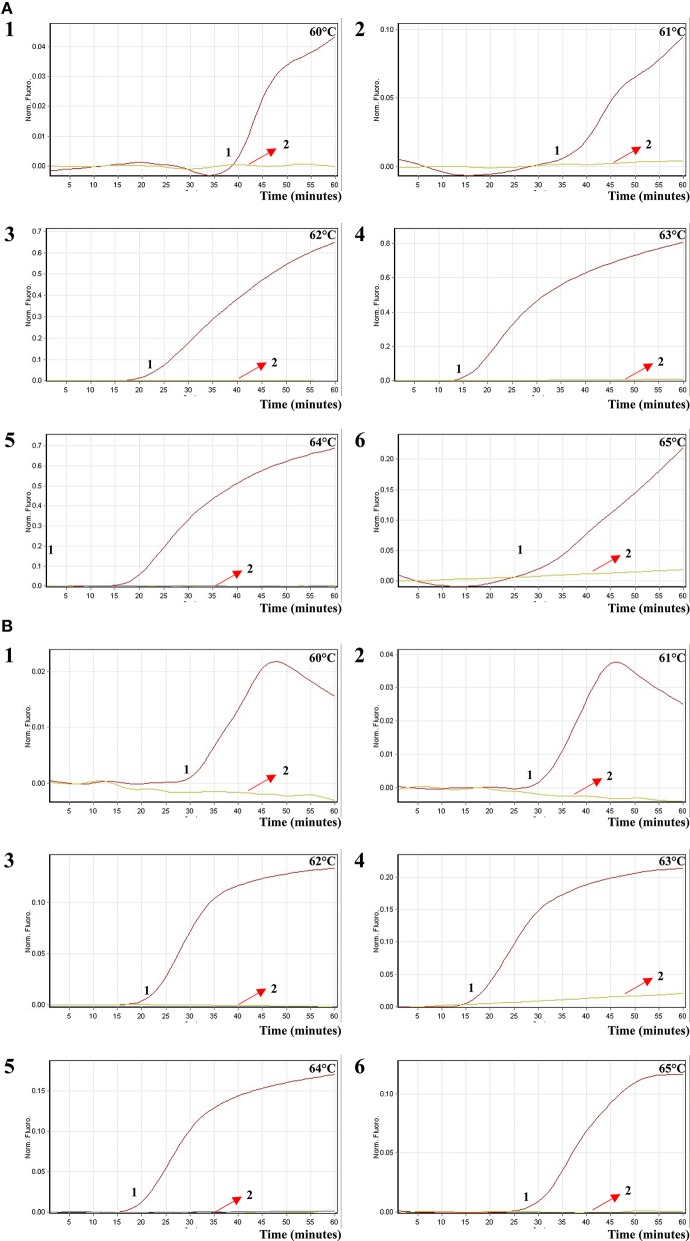
**The optimal reaction temperature of the ET-MCDA assay**. Two sets of ET-MCDA primers targeting *invA* and *ipaH* genes were used in the same reaction tube, **(A,B)** were simultaneously generated from Cy5 (labeling *Sal*-E-CP1 of *invA*) and Hex (labeling *Shi*-E-CP1 of *ipaH*) channels, respectively. The multiplex ET-MCDA amplifications were monitored by means of real-time format, and the corresponding curves of DNA concentrations were listed. Signal 1 indicates *Salmonella enterica* serovar *Enteritidis* strains of in Cy5 channel **(A)**, *Shigella flexneri* strain for Hex channel **(B)**, and signal 2 indicates negative control. Six kinetic graphs (1–6) were produced at different reaction temperature (60–65°C at 1°C intervals) with *Salmonella* genomic DNA at the level of 250 pg in Cy5 channel **(A)**; another six kinetic graphs (1–6) were obtained at different detection temperature (60–65°C at 1°C intervals) with *Shigella* genomic DNA at the level of 250 pg in Hex channel **(B)**. The graphs from 62°C to 64°C displayed robust amplification.

In order to test the optimal amplification temperature of the ET-MCDA method, the ET-MCDA reactions, which simultaneously contained the *Salmonella* and *Shigella* genomic templates at the level of 250 pg per tube, were performed at various temperatures (60–65°C) with 1°C intervals according to the multiplex ET-MCDA conditions. The results were detected by means of real-time format, and the typical kinetics graphs were yielded (Figure 5). Each amplification temperature provided a robust signal corresponding to Cy5 and Hex channel, with the faster amplification seen for method temperature 62–64°C, which were recommended as the standard detection temperature for ET-MCDA technique. In our study, the reaction temperature of 63°C, which achieved rapid determination of target sequences with better fluorescence signals, was selected for the rest of ET-MCDA performance conducted in this study.

### Real-time detection of a single target in an ET-MCDA reaction

**Figure 6:**
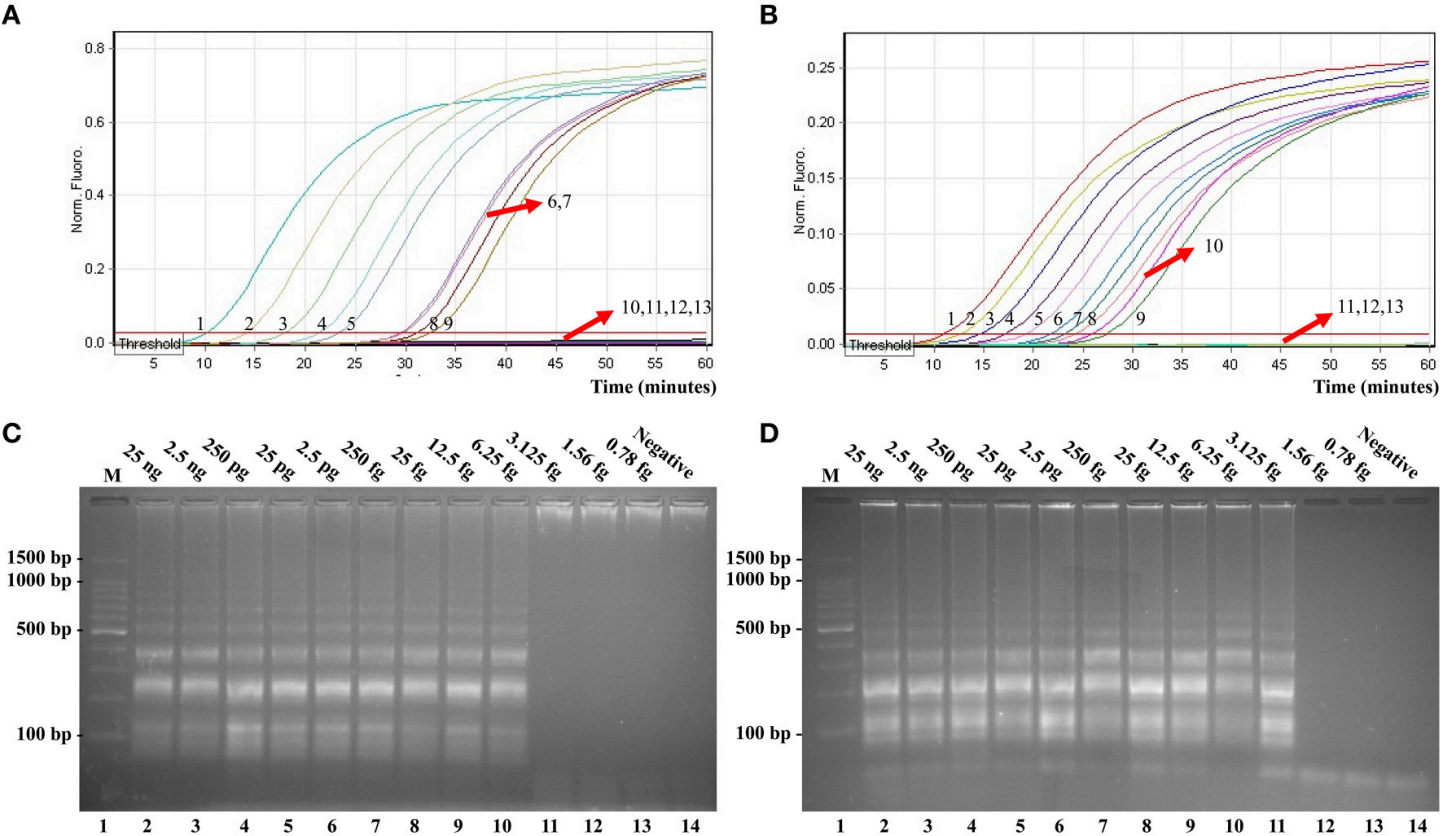
**Real-time detection of a single target in an ET-MCDA**. Analytical sensitivity of *Salmonella*-ET-MCDA **(A)** and *Shigella*-ET-MCDA **(B)** for *Salmonella* and *Shigella* detection was analyzed by real-time format, and signals 1, 2, 3, 4, 5, 6, 7, 8, 9, 10, 11, 12, and 13 represent DNA levels of 25 ng, 2.5 ng, 250 pg, 25 pg, 2.5 pg, 250 fg, 25 fg, 12.5 fg, 6.25 fg, 3.125 fg, 1.56 fg, and 0.78 fg per tube and negative control. The LoD of *Salmonella*-ET-MCDA approach was 6.25 fg per tube, and the *Shigella*-ET-MCDA for 3.125 fg per tube. Analytical sensitivity of *Salmonella*-ET-MCDA **(C)** and *Shigella*-ET-MCDA **(D)** for *Salmonella* and *Shigella* detection was also analyzed by 2% agarose gel electrophoresis, and the positive reactions were observed as a ladder-like pattern on 2% agarose gel electrophoresis analysis. Lane 1, DL 100-bp DNA marker.

In this report, we assessed ET-MCDA detection in a single target format by using separate amplification of *invA* sequence (*Salmonella*-specific gene) and *ipaH* sequence (*Shigella*-specific gene) from a *Salmonella* and *Shigella* genomic template with E-CP1 in each reaction. As expected, the release of quenching was seen as a robust increase of Cy5 and Hex signals, and positive reactions were produced in approximately 12 min (Figures 6A,B). The LoD of ET-MCDA assay for independently analyzing *invA* gene and *ipaH* gene was 6.25 and 3.125 fg genomic DNA templates per tube, respectively (Figures 6A,B). Moreover, the reaction products were also analyzed by 2% agarose gel electrophoresis, and the typical ladder-like patterns were seen in positive amplifications but not in negative amplification and control (Figures 6C,D). The LoD of the real-time measurement for *Salmonella*- and *Shigella*-ET-MCDA detections was in conformity with agarose gel electrophoresis analysis.

**Figure 7:**
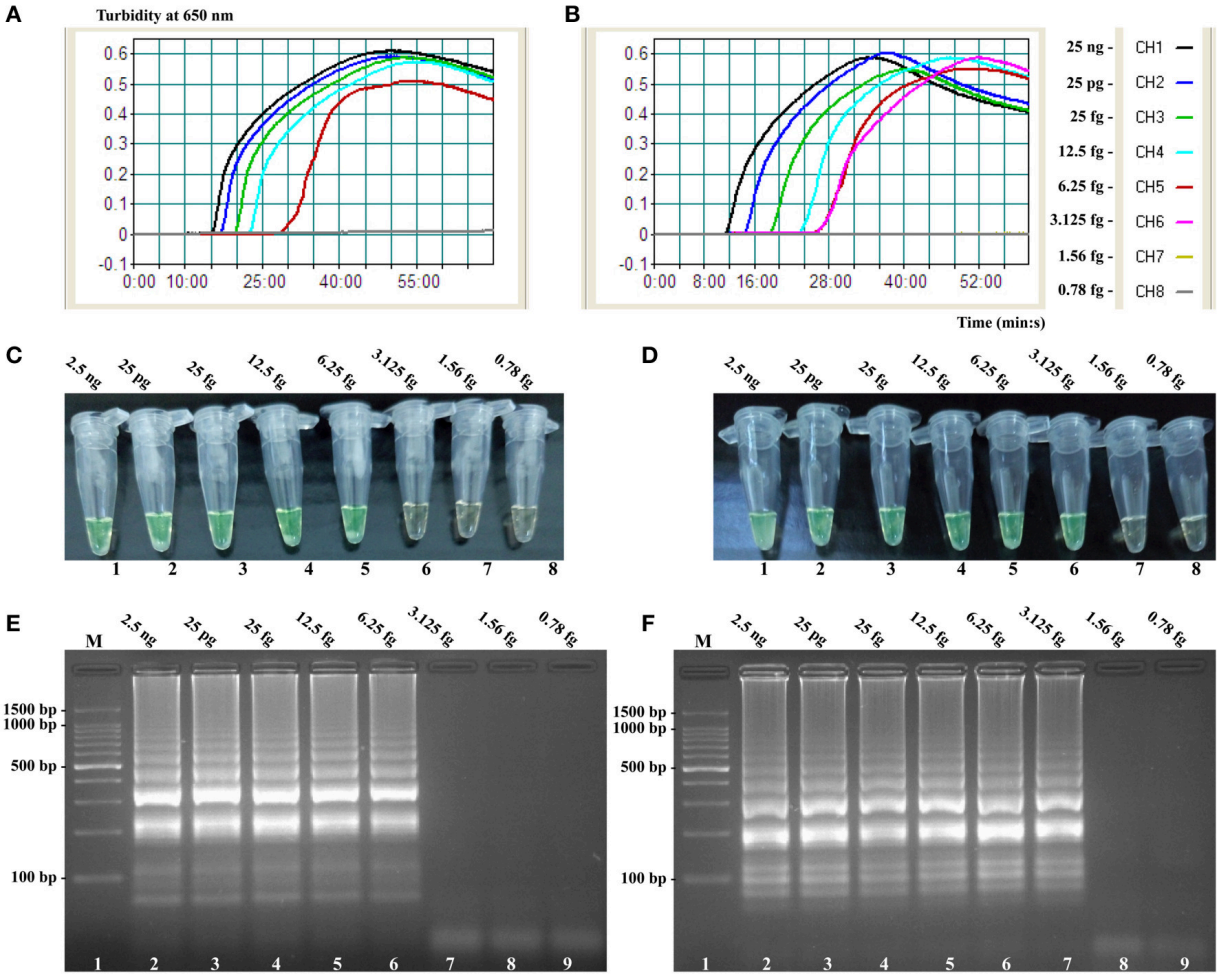
**Sensitivity of the *invA*- and *ipaH*-MCDA techniques using serially genomic DNA with *Salmonella* strains and *Shigella* strains as templates**. Sensitivity of *invA*-MCDA for *Salmonella* **(A)** and *ipaH*-MCDA for *Shigella* **(B)** detection was monitored by real-time measurement of turbidity and the corresponding curves of concentrations of genomic DNA were marked in the figure. The LoD of *invA*-MCDA assay was 6.25 fg per vessel, and the *ipaH*-MCDA for 3.125 fg per reaction. Sensitivity of *invA*-MCDA for *Salmonella* **(C)** and *ipaH*-MCDA for *Shigella* **(D)** detection was monitored by FD reagent, and a color shift of positive reactions in *Salmonella*- and *Shigella*-MCDA tubes from light gray to green were directly seen with naked eyes. Moreover, the sensitivity of *invA*-MCDA for *Salmonella* **(E)** and *ipaH*-MCDA for *Shigella* **(F)** detection was analyzed by 2% agarose gel electrophoresis, and the positive results were seen as a ladder-like pattern on 2% agarose gel electrophoresis analysis. Lane 1, DL 100-bp DNA marker.

The LoD of ET-MCDA, MCDA, qPCR, and PCR approaches on *Salmonella* strains was 6.25 fg, 6.25 fg, 2.5 pg, and 25 pg per reaction, and on *Shigella* strains was 3.125 fg, 3.125 fg, 2.5 pg, and 25 pg per reaction, respectively (Figures 6, 7, Table 3). These results demonstrated that the analytical sensitivity of ET-MCDA methodology for identifying a single target was in complete accordance with MCDA assay, whereas was at least 400- and 4000-flod more sensitive than that of qPCR and PCR techniques, respectively.

**Table 3:** **The sensitivity and time for single ET-MCDA targeting *invA* and *ipaH* genes compared with that of qPCR and conventional PCR techniques**.

**Techniques^a^**	**Isothermal amplification**	**Regions recognized**	**Multiplex detection**	**LoD for *Salmonella* spp. *Shigella* spp. (no./reaction)**	**Fastest time (minutes)**	**LoD time (minutes)^b^**
ET-MCDA	+	10	+	6.25 fg/3.125 fg	12	35
MCDA	+	10	−	6.25 fg/3.125 fg	12	35
qPCR	−	3	+	2.5 pg/2.5 pg	32	56
PCR	−	2	+	25 pg/25 pg	150	150

a *ET-MCDA, endonuclease restriction-mediated real-time multiple cross displacement amplification; MCDA, multiple cross displacement amplification; qPCR, quantitative real-time PCR*.

b *LoD, limit of detection. The LoD values are the lowest gnomic DNA level that was positively amplified in triplicate. The positive results of qPCR were generated as c_t_ values, which converted to time for detection*.

### Real-time detection of multiple targets in an ET-MCDA reaction

**Figure 8:**
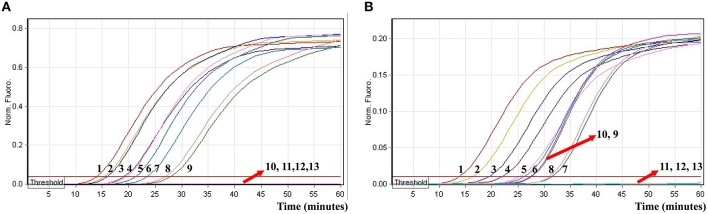
**The real-time analysis of multiplex targets in an ET-MCDA reaction**. Two sets of ET-MCDA primers targeting *invA* and *ipaH* genes were simultaneously added to a reaction tube. **(A,B)** were simultaneously yielded from Cy5 (labeling *Sal*-E-CP2 of *invA*) and Hex (labeling *Shi*-E-CP1 of *ipaH*) channels, respectively. The analytical sensitivity of multiplex-ET-MCDA for simultaneously detecting *Salmonella* **(A)** and *Shigella* **(B)** was analyzed by real-time format, and signals 1, 2, 3, 4, 5, 6, 7, 8, 9, 10, 11, 12, and 13 represent DNA levels of 25 ng, 2.5 ng, 250 pg, 25 pg, 2.5 pg, 250 fg, 25 fg, 12.5 fg, 6.25 fg, 3.125 fg, 1.56 fg, and 0.78 fg per tube and negative control. The LoD of multiplex ET-MCDA assay for *Salmonella* detection was 6.25 fg per reaction, and the LoD of multiplex ET-MCDA for *Shigella* detection was 3.125 fg per reaction.

In order to evaluate the capability of ET-MCDA for simultaneously detecting multiple targets, the novel ET-MCDA technique was applied to detect and differentiate two targets in a single reaction. In order to facilitate multiplex detection, we modified the amount of the primers on the base of standard ET-MCDA system, and the multiplex ET-MCDA reactions were also conducted at 63°C for 1 h. Under the multiplex conditions, two different fluorescence curves were simultaneously obtained from multiplex ET-MCDA reactions containing two complete primer sets and their corresponding genomic templates (Figure 8). The ET-MCDA technology successfully detected and distinguished *Salmonella* and *Shigella* in a single reaction, and simultaneously provided two sets of robust signals for two targets. The analytical sensitivity of ET-MCDA approach for simultaneously detecting *invA* and *ipaH* genes was 6.25 and 3.125 fg of each genomic DNA templates per reaction, respectively (Figure 8). No difference of LoD was observed between detecting a single target and multiple targets in ET-MCDA methodology.

### Analytical specificity of the ET-MCDA technology

**Figure 9:**
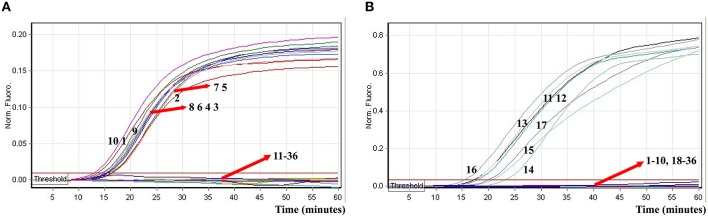
**The analytical specificity of multiplex ET-MCDA detection for different strains**. The multiplex ET-MCDA amplifications were conducted using different genomic DNA templates and were monitored by means of real-time format. **(A,B)** were simultaneously generated from HEX and Cy5 channels. Signals 1-10, *Shigella flexneri* strains of serovar 1d (ICDC-NPS001), 4a (ICDC-NPS002), 5a (ICDC-NPS003), 2b (ICDC-NPS004), 1b (ICDC-NPS005), 3a (ICDC-NPS006), 3b (ICDC-NPS008), *Shigella boydii, Shigella sonneri*, and *Shigella dysenteriae*; signals 11–15, *Salmonella Choleraesuis* (ICDC-NPSa001), *Salmonella Dublin* (ICDC-NPSa002), *Salmonella Enteritidis* (ICDC-NPSa003), *Salmonella Typhimurium* (ICDC-NPSa004), *Salmonella Weltevreden* (ICDC-NPSa005); signals 16 and 17, two *Salmonella* strains of unidentified serotype; signals 18-35, *Listeria monocytogenes* stains of serovar 1/2a (EGD-e), 4c (19116), *Enteroinvasive E. coli, Enteropathogenic E. coli, Enterotoxigenic E. coli, Enteroaggregative E. coli, Enterohemorrhagic E. coli, Plesiomonas shigelloides, Yersinia enterocolitica, Bntorobater sakazakii, Enterobacter cloacae, Enterococcus faecalis, Vibrio parahaemolyticus, Vibrio vulnificus, Bacillus cereus, Staphylococcus aureus, Campylobacter jejuni*, and *Pseudomonas aeruginosa*, signal 36, negative control.

In order to determine analytical specificity of the novel ET-MCDA technique, we carried out the multiplex ET-MCDA amplifications under the multiplex conditions described above with the purely genomic DNA templates extracted from 20 *Shigella*, 15 *Salmonella*, and 20 non-*Shigella* and non-*Salmonella* strains (Table 2). The positive results were obtained only when genomic DNAs of *Shigella* and *Salmonella* isolates were used as templates in multiplex ET-MCDA reaction, and the non-*Salmonella*, non-*Shigella* and negative control examined by multiplex ET-MCDA system were negative after 1 h incubation period (Figure 9). Moreover, the target pathogens could be simultaneously detected and correctly differentiated in the same reaction tube. These results suggested that the novel ET-MCDA technique reported here was specific to target sequence identification.

### Evaluation of the ET-MCDA technology by using artificially contaminated blood samples

**Figure 10:**
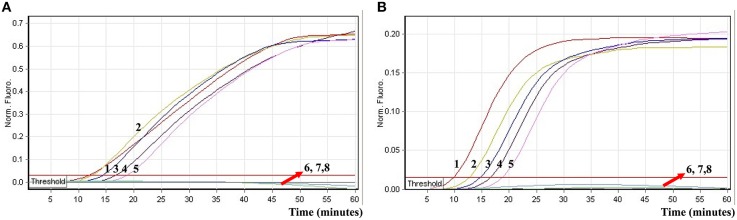
**The sensitivity of multiplex ET-MCDA approach for simultaneously detecting two target pathogens in artificially contaminated blood samples**. Two sets of ET-MCDA primers targeting *invA* and *ipaH* genes were simultaneously added to a reaction vessel. **(A,B)** were simultaneously yielded from Cy5 (labeling *Sal*-E-CP1 of *invA*) and Hex (labeling *Shi*-E-CP1 of *ipaH*) channels, respectively. Sensitivity of multiplex-ET-MCDA for simultaneously detecting *Salmonella* **(A)** and *Shigella* **(B)** in artificially contaminates blood samples was monitored by real-time format, and signals 1, 2, 3, 4, 5, 6, 7, and 8 represent *Salmonella* DNA levels of 39,000, 3900, 390, 39, 3.9, 0.39, and 0.039 CFU per reaction and negative control; *Salmonella* DNA levels for 34,000, 3400, 340, 34, 3.4, 0.34, and 0.034 CFU per reaction and negative control. The LoD of multiplex ET-MCDA method for *Salmonella* analysis in artificially contaminates blood samples was 3.9 CFU per reaction, and the LoD of multiplex ET-MCDA for *Salmonella* detection in artificially contaminates blood samples was 3.4 CFU per reaction.

To further validate the feasibility of the ET-MCDA technology as a nucleic acid analysis tool, the novel ET-MCDA assay was examined by the artificially adding *Salmonella* strain and *Shigella* strain into blood samples. The multiplex ET-MCDA technology could generated positive result when the contaminated numbers of *Shigella* and *Salmonella* were more than 3.9 × 10^2^ CFU/ml (~3.9 CFU/tube) and 3.4 × 10^2^ CFU/ml (~3.4 CFU/tube), respectively, and the two target pathogens were simultaneously detected and correctly identified in a single ET-MCDA reaction (Figure 10 and Table 4). The non-contaminated blood sample and negative control was found to be negative.

**Table 4:** **Comparison of ET-MCDA, MCDA, qPCR, and PCR technique for detection of *Salmonella* spp. and *Shigella* spp. in artificially contaminated blood samples**.

**Detection technique^a^**	**Multiplex detection**	**LoD (no./reaction)**	
		***Salmonella* spp. detection**	***Shigella* spp. detection**
ET-MCDA	+	3.9 CFU ~ (3.9 × 10^2^ CFU/ml)	3.4 CFU ~ (3.4 × 10^2^ CFU/ml)
MCDA	−	3.9 CFU ~ (3.9 × 10^2^ CFU/ml)	3.4 CFU ~ (3.4 × 10^2^ CFU/ml)
qPCR	−	39 CFU ~ (3.9 × 10^3^ CFU/ml)	34 CFU ~ (3.4 × 10^3^ CFU/ml)
PCR	−	390 CFU ~ (3.9 × 10^4^ CFU/ml)	340 CFU ~ (3.4 × 10^4^ CFU/ml)

*^a^ET-MCDA, endonuclease restriction-mediated real-time multiple cross displacement amplification; MCDA, multiple cross displacement amplification*.

The LoD of multiplex ET-MCDA technique was identical with that of normal MCDA detection only for *Salmonella* or *Shigella* strains in artificially contaminated blood samples, respectively (Table 4). In contrast, the qPCR and PCR assays produced positive results when the contaminate numbers of *Salmonella* for 3.9 × 10^3^ CFU/ml (~39 CFU/tube) and 3.9 × 10^4^ CFU/ml (~390 CFU/tube), *Shigella* amounted to more than 3.4 × 10^3^ CFU/ml (~34 CFU/tube) and 3.4 × 10^4^ CFU/ml (~340 CFU/tube), respectively. The results demonstrated that the LoD of multiplex ET-MCDA was 10- and 100-fold more sensitive than that of qPCR and PCR approaches (Table 4).

## Discussion

Isothermal amplification of the nucleic acids, which has been validated as a simple, rapid and efficient process, could specifically accumulate the target sequences at a single temperature (Zhao et al., 2015). Herein, numerous isothermal nucleic acids amplification technologies employing various amplification mechanisms have been established, which have been used as valuable molecular tools in basic research, medical diagnosis, epidemiology and many other fields. However, their uptake in the field of multiplex detection has been restricted, limiting the wider usefulness of these methodologies (Jodi Woan-Fei et al., 2015). Previous reports have devised several strategies for the multiplex isothermal detection, while these assays required special reagents, further processing and apparatus, and did not allow real-time detection (Wang et al., 2015a). Therefore, the development of alternative techniques for simple, rapid, efficient and multiplex isothermal amplification detection is in continuous demand.

This report represented here describes the development of the novel endonuclease restriction-mediated real-time multiple cross displacement amplification (ET-MCDA), which integrated isothermal amplification approach, real-time and fluorescence detection technology. The key feature of the ET-MCDA technique was use of the endonuclease recognition site at the 5′ end and fluorophore labeling of distinct targets, and use of the endonuclease restriction (Nb.*BsrDI*) for specially cleaving double-stranded sequences to release quenching, resulting in a gain of fluorescence signal. Thus, the novel ET-MCDA technology facilitated rapid detection and simultaneous identification of multiple different targets in only one isothermal step, eliminating the use of temperature-regulating equipment and the use of further post detection analysis. In the course of the ET-MCDA reaction, the amplification vessels were not opened, which effectively alleviated any carryover contamination. Moreover, the ET-MCDA methodology could offer real-time detection of single or multiple targets in a single tube, and positive results could be generated in as short as 12 min. Considering these traits, the ET-MCDA technique can be a valuable tool for devising handheld diagnostic devices, which could be used for detecting nucleic acids in various fields, such as field testing, clinical and point-of-care diagnosis, and more.

As a proof of concept, two important pathogens (*Shigella* and *Salmonella*), which were the most frequent bacterial causes of dysentery worldwide, were selected as models for illustrated the capability of singlex or multiplex ET-MCDA detection (Pfeiffer et al., 2012). First, we tested the ET-MCDA method in a single target format by using separate detection of *Shigella* spp.-specific gene (*ipaH*) and *Salmonella* spp.-specific gene (*invA*) with *Shigella* and *Salmonella* purely genomic templates, respectively. The analytical sensitivity of ET-MCDA approach for independently identifying *Shigella* and *Salmonella* strains was 3.125 and 6.25 fg pure DNA templates per reaction, respectively, which was consistent with the normal MCDA method (Figures 6, 7). However, the ET-MCDA technology performed better than qPCR assay with respect to detection speed and LoD. The novel ET-MCDA technique was at least 400-fold more sensitive than qPCR approach, and was faster than qPCR assay approximately 20 min, resulting in shortening total assay time (Table 3). Comparing with traditional PCR method, the ET-MCDA technique, obviating the use of agarose gel electrophoresis analysis, was at least 4000-fold more sensitive than PCR assay for detecting pure templates. The *Salmonella*-qPCR, *Salmonella*-PCR, *Shigella*-qPCR, and *Shigella*-PCR methods have been reported in previous studies, which were employed to ascertained the LoD of qPCR and PCR techniques (Daum et al., 2002; Fratamico, 2003; Aranda et al., 2004; Vu et al., 2004).

In this study, the multiplex ET-MCDA reactions also showed the robust amplification of the two targets, which successfully detected *Shigella* and *Salmonella* in a single tube, and simultaneously produced two sets of robust signals for target pathogens (Figure 8). The analytical sensitivity of ET-MCDA assays for simultaneously detecting *Shigella* and *Salmonella* strains was 3.125 and 6.25 fg of each genomic templates per reaction, respectively, which was identical to that of singlex ET-MCDA reaction (Figures 6, 8). No difference of LoD was observed between detecting a single target and multiple targets in an ET-MCDA system. Moreover, the analytical sensitivity of multiplex ET-MCDA reactions was also the same as that of ordinary MCDA assay, but the novel ET-MCDA technique could simultaneously identify and distinguish multiple targets.

A set of 10 sequence-specific primers, which recognized 10 regions (F1, F2, C1, C2, P1, P2, R1, R2, D1, and D2) spanning more than 200 bp for specific detection of per target, provided a high degree of specificity (Wang et al., 2015d). For the ET-MCDA technique specificity test, positive results were produced from *Shigella* and *Salmonella* strains, and no positive signals were observed in the assay of non-*Shigella* and non-*Salmonella* strains (Figure 9). Moreover, the novel ET-MCDA approach established here could correctly differentiate target sequences in only one isothermal step with easily interpretable results.

In order to evaluate the practical application of ET-MCDA detection of *Shigella* strains and *Salmonella* strains in clinical samples, artificially contaminated blood samples were analyzed by ET-MCDA, MCDA, qPCR, and PCR assays. The LoD of ET-MCDA for *Shigella* strains and *Salmonella* strains detection in artificially contaminated blood samples was 3.4 CFU and 3.9 CFU per reaction, which was also in conformity with ordinary MCDA analysis (Table 4). However, the two target pathogens could be simultaneously detected within 40 min with only one isothermal amplification step and the positive reactions were displayed in real-time format (Figure 10). Furthermore, the analytical sensitivity of ET-MCDA methodology for artificially contaminated blood samples was 10- and 100-fold more sensitive than qPCR and PCR approaches. As such, the novel ET-MCDA technique was more suitable than MCDA, qPCR, and PCR methods for multiplex, rapid, sensitive and specific detection of *Shigella* strains and *Salmonella* strains in practical samples.

In conclusion, a novel ET-MCDA technology developed here, which integrated multiple cross displacement amplification strategy, restriction endonuclease cleavage and real-time fluorescence detection technique, facilitated multiplex, rapid, specific and sensitive detection of nucleic-acid sequence at a constant temperature. The ET-MCDA assay had the advantages over the PCR-based approaches, namely, modest equipment requirements, quick results, easy operation, cost and energy efficiency, high specificity and sensitivity, thus these attractive traits will motivate the researchers to explore the wider application of the novel technology for nucleic acid analysis in various fields.

## Author contributions

Conceived and designed the experiments: YiW, JX, and CY. Performed the experiments: YiW, YaW, LZ, DL, LL, HL, XC, and KL. Analyzed the data: YiW and YaW. Contributed reagents/materials/analysis tools: YiW, YaW, LZ, DL, LL, HL, XC, and KL. Designed the software used in the analysis: YiW. Wrote the manuscript: YiW, JX, and CY.

## Funding

We acknowledge the financial supports of the grants (Mega Project of Research on the Prevention and Control of HIV/AIDS, Viral Hepatitis Infectious Diseases 2011ZX10004-001, 2013ZX10004-101 to CY) from the Ministry of Science and Technology, People's Republic of China, and grant (2015SKLID507 to CY) from State Key Laboratory of Infectious Disease Prevention and Control, China CDC.

## Disclosures

YiW and CY have filed for a patent from the State Intellectual Property Office of China, which covers the novel technique and sequences included in the study (Application number CN 201610219350.6).

### Conflict of interest statement

The authors declare that the research was conducted in the absence of any commercial or financial relationships that could be construed as a potential conflict of interest.
